# Exploration of machine learning techniques in predicting multiple sclerosis disease course

**DOI:** 10.1371/journal.pone.0174866

**Published:** 2017-04-05

**Authors:** Yijun Zhao, Brian C. Healy, Dalia Rotstein, Charles R. G. Guttmann, Rohit Bakshi, Howard L. Weiner, Carla E. Brodley, Tanuja Chitnis

**Affiliations:** 1 Department of Computer Science, Tufts University, Medford, Massachusetts, United States of America; 2 Partners MS Center, Brigham and Women’s Hospital, Brookline, Massachusetts, United States of America; 3 Biostatistics Center, Massachusetts General Hospital, Boston, Massachusetts, United States of America; 4 College of Computer and Information Science, Northeastern, Boston, Massachusetts, United States of America; University of Oxford, UNITED KINGDOM

## Abstract

**Objective:**

To explore the value of machine learning methods for predicting multiple sclerosis disease course.

**Methods:**

1693 CLIMB study patients were classified as increased EDSS≥1.5 (worsening) or not (non-worsening) at up to five years after baseline visit. Support vector machines (SVM) were used to build the classifier, and compared to logistic regression (LR) using demographic, clinical and MRI data obtained at years one and two to predict EDSS at five years follow-up.

**Results:**

Baseline data alone provided little predictive value. Clinical observation for one year improved overall SVM sensitivity to 62% and specificity to 65% in predicting worsening cases. The addition of one year MRI data improved sensitivity to 71% and specificity to 68%. Use of non-uniform misclassification costs in the SVM model, weighting towards increased sensitivity, improved predictions (up to 86%). Sensitivity, specificity, and overall accuracy improved minimally with additional follow-up data. Predictions improved within specific groups defined by baseline EDSS. LR performed more poorly than SVM in most cases. Race, family history of MS, and brain parenchymal fraction, ranked highly as predictors of the non-worsening group. Brain T2 lesion volume ranked highly as predictive of the worsening group.

**Interpretation:**

SVM incorporating short-term clinical and brain MRI data, class imbalance corrective measures, and classification costs may be a promising means to predict MS disease course, and for selection of patients suitable for more aggressive treatment regimens.

## Introduction

A critical component in the management of patients with multiple sclerosis (MS) is correctly predicting which patients will experience worsening disease over the short term. This is particularly relevant given the expanding array of disease-modifying medications, and the importance of identifying the patients who may benefit from more potent or aggressive treatment or closer monitoring. Although a number of clinical and demographic features have been associated with long-term disease course in MS,[1–7] prediction of disease course from demographic and/or baseline clinical data is challenging, and no validated prediction model for disease worsening is currently available. In this paper we explore both logistic regression and machine-learning techniques in predicting disease course and their relative performance using baseline data or longitudinal data. A key question that we address is the length of monitoring period required for the best model performance.

Logistic regression is a statistical method for finding the best fitting linear relationship between the log odds of a binary variable (“worsening” versus “non-worsening” in our case) and a group of independent explanatory variables (patients’ longitudinal records in our case). Support Vector Machine (SVM) is a widely used machine-learning classification method where the algorithm maximizes the margin that separates the two classes of data.

Even though traditional logistic regression and SVM can be applied to develop prediction models, MS datasets including ours have additional features that require attention. First, because the prevalence of patients with worsening disease is smaller than those with a milder course, we have a skewed distribution, often called class imbalance.[8] Second, incorrectly classifying subjects who will experience worsening disease incurs a higher cost because the consequences of leaving a patient with worsening disease inadequately treated are potentially worse than the side effects that may be involved in aggressively treating a patient with relatively mild disease. Third, the amount of longitufdinal follow-up required for good clinical prediction is uncertain. In this paper, we assess extensions to traditional prediction models to account for the complexities of MS datasets in order to obtain improved performance.

## Materials and methods

### Study overview

The Comprehensive Longitudinal Investigation of MS at the Brigham and Women’s Hospital, Partners MS Center (CLIMB) has been enrolling patients since 2000. Patients meeting the following inclusion criteria were included in this study: 1) subjects with a visit at year 5 2) 2) at least three clinical visits on the study in order to contribute 3) smoking history; (Table 1). The basic schema of this study is described in prior publications.[9, 10]

**Table 1:** Baseline demographic and clinical characteristics of study sample.

N	1693
Number of females (%)	1248 (73.7%)
Age [years, mean (SD)]	43.88 (11.46)
Number self-reported white (%)	1562 (92.3%)
EDSS (≤1.5 **/** 2–4 / ≥4)	919 / 539 / 235

EDSS-Expanded Disability Status Scale

*Clinical data collection*: CLIMB patients have a complete neurological exam every six months that includes measurement of the Expanded Disability Status Scores (EDSS).

*MRI segmentation*: All baseline and follow-up brain scans from our institution were processed by a semi-automated template driven segmentation tool, as previously described.[11] Here, we include annual measurements of whole brain T2 hyperintense lesion volume (T2LV) and normalized whole brain volume, expressed as the brain parenchymal fraction [BPF = (gray matter+white matter+T2LV)/intracranial volume].

### Subject selection

**Fig 1:**
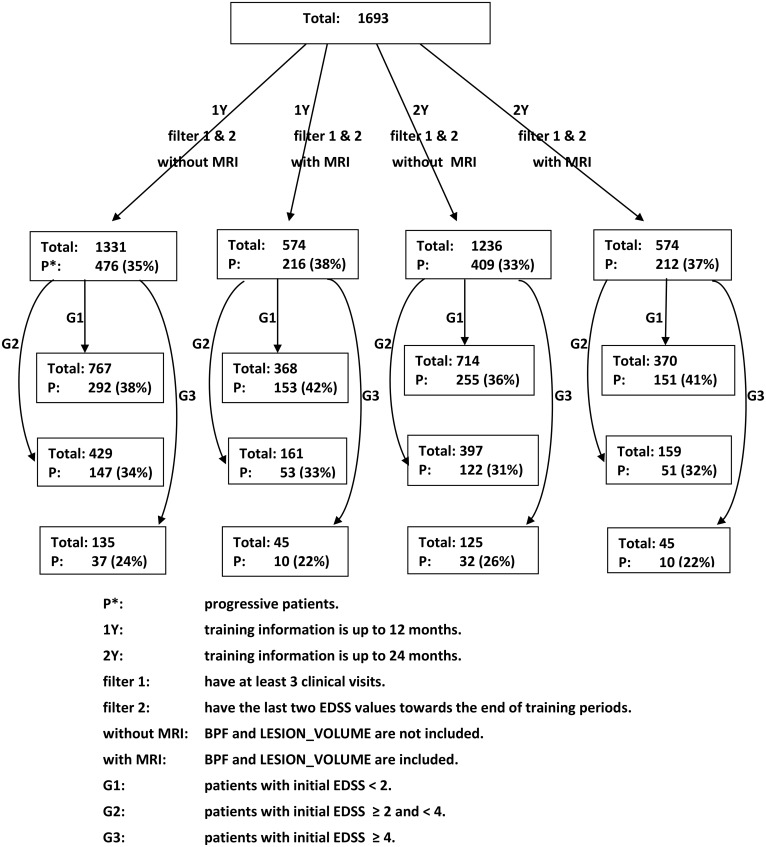
Flowchart of patient selection. Fig 1 presents the distribution of patients after imputing the missing values. The labels on the arrows indicate the number of years of follow-up required in the training dataset and which filters were applied. The first box indicates the total number of patients assessed. Note that P (and %P) refers to the number (and percentage) of patients who meet “progression” criteria within the different subgroups.

We selected subgroups of patients by applying various filters such as length of observation (baseline, one year and two years) and with/without MRI information. The flowchart in Fig 1 outlines details of each filter and the number of patients included in each subgroup. Note that when applying our methods to one and two year data, we further filtered to remove patients with many missing visits (patients miss visits for a variety of reasons which include travel, forgotten appointments, insurance issues). The CLIMB study is approved by the Partners Institutional Review Board. Subjects provided written consent for participation in the CLIMB study.

### Statistical analysis/Machine learning approach

#### Overview of machine learning

We investigated two different learning algorithms: Logistic regression and Support Vector Machines (SVM), and the impact of linear versus non-linear kernels for SVMs on performance [12–14]. In addition to examining the length of time from which to make a prediction, we investigated several modifications to these two basic approaches designed to handle the complexities of the data: non-uniform misclassification costs, class imbalance and missing data. We describe each in turn.

#### Classification models for EDSS outcomes

Our main outcome in these experiments is “worsening” or “non-worsening” EDSS at five years of follow-up from entry into the study. We define “worsening” as an increase of 1.5 or more from baseline EDSS to EDSS at the five year mark, and “non-worsening” as all other cases, based on the fact that EDSS increase of 1.0 or 1.5 a clinically significant and generally sustained increase in EDSS, and is used as a primary or secondary endpoint in clinical trials of MS therapies.

We estimate the predictive accuracy of models for “worsening” versus “non-worsening” MS at 5 years of follow-up using: 1) data available from the initial visits, 2) information available up to the 12-month clinical visit, and 3) information available up to the 24-month visit. For each longitudinal dataset, in addition to including the cross-sectional clinical information from each visit, we created new features that described the change in clinical information between visits.

#### Baseline groups

Because patients with a different baseline EDSS score may have different disease trajectories, we created categories of patients by initial EDSS score: G1-initial EDSS<2; G2-initial EDSS> = 2 and <4; G3-EDSS> = 4.

#### Predictors of disease course

Potential predictors included in the models are listed in Table 2. These include demographic characteristics, clinical characteristics, MRI features, and characteristics of the first symptom(s) of MS. Several of these features have been identified in previous papers as predictive of long-term outcomes in patients with MS.[2, 6, 15] Because of the inclusion of longitudinal data, we further investigate whether the changes in some measures lead to improved predictive accuracy.

**Table 2:** Predictors of disease classification.

Demographic	Visit age Disease duration at baseline visit Gender Race Ethnicity Family history of MS Smoking ever
Clinical	EDSS Ambulation Index Disease step Disease category Disease activity Pyramidal_functional status score Cerebellar_functional status score Brainstem_functional status score Sensory_functional status score Bowel_bladder_functional status score Visual functional status score Mental functional status score
MRI	BPF T2 lesion volume
Additional predictors	**Change** of each clinical and MRI value over its corresponding initial value

EDSS-Expanded Disability Status Scale, FS-functional status, BPF-brain parenchymal fraction

#### Handling missing data

In order to handle missing data, we model each patient’s record as a time series with each clinical visit as one data point. Missing numeric values in the time series are interpolated/extrapolated linearly using the nearest data points. Missing categorical values are filled using the mode of existing values in the patient’s time series. Each time series has a potential total of eleven time points given that patients have an initial visit plus follow-up every six months visits for up to five years. We required each time series to have at least three data points (out of eleven). Records with fewer than three data points in the time series are removed from the experiment (Fig 1).

#### Class imbalance

In this dataset, there are more patients in the “non-worsening” EDSS class than the “worsening” EDSS class. Thus we perform a combination of bagging and undersampling[8] of the majority class during training. Specifically, we form ten classifiers, each trained on all minority class (“worsening” EDSS) instances and a random sample of equal number of the majority class (“non-worsening” EDSS) instances. When predicting the class of a previously unseen instance, we take a majority vote of the ten classifiers.

#### “Cost” of misclassification

Our last consideration is the relative costs of false positives (predicting progressive when a patient has non-progressive EDSS) and false negatives (predicting non-progressive when a patient has progressive EDSS). To address this misclassification cost disparity, we ran the SVM method first with equal misclassification costs and then with a cost ratio of *c*:1, where c ranges from 1.5 to 3 with an increment of 0.5 (thus misclassifying progressive as non-progressive EDSS was *c* times as costly as the reverse). When forming a classifier, the SVM optimizes a function of cost rather than accuracy.[16]

#### Reporting of data

The sensitivity, specificity, and overall accuracy of the model are reported for each approach and model. Sensitivity is defined in this study as the proportion of subjects who worsen that are correctly classified as worsening. Specificity is defined as the proportion of subjects who did not worsen that are correctly classified. Overall accuracy is defined as the proportion of all subjects who are correctly classified. Each outcome measure was calculated based on a ten-fold cross-validation for each of the experiments. Ten-fold cross-validation breaks the data into ten parts. The model is built using 90% of the data and then tested in the remaining 10%, and this procedure is repeated for each of the ten parts of the data. Since each observation is left out of the model building one time, the sensitivity, specificity and accuracy associated with each observation is calculated based on this aspect of the cross-validation.

*Software*: All analyses were completed using the machine learning software package Weka 3.4.

## Results

### Prediction based on baseline data only

We first assessed whether clinical information including EDSS scores, FS scores, and demographic features could accurately predict whether at patient would be progressive or non-progressive at 5 years. Using a sample of 1352 patients, 525 of whom were progressive at 5 years, we attained an overall accuracy rate for logistic regression of 62% and for linear SVM of 64% (SVM with a non-linear kernel fared no better (Table 3)). The issue with these baseline methods is that they are heavily weighted toward always saying a patient is non-progressive (accuracies of 84% ad 91% for LR and SVM respectively). Since there are more non-progressive patients than progressive patients, these levels of accuracy would not be considered useful in clinical practice. When baseline MRI information of T2 lesion volume and BPF was included, the overall accuracy improved to 68% and 70% for LR and SVM respectively. However, the predictive accuracy of the progressive group still remains lower than for the non-progressive group for all models. Finally, we observe that among LR and the three variants of SVM, there is little to no difference in performance.

**Table 3:** Predictive accuracy for different groups using 12 month visit without MRI based on ten-fold cross-validation.

Method	Sensitivity				Specificity				Overall Accuracy			
	G0	G1	G2	G3	G0	G1	G2	G3	G0	G1	G2	G3
logistic +bagging	0.62	0.56	0.55	0.54	0.64	0.64	0.67	0.64	0.63	0.61	0.63	0.61
logistic	0.35	0.41	0.38	0.54	0.86	0.83	0.79	0.64	0.68	0.67	0.65	0.61
SVM+bagging	0.62	0.53	0.66	0.53	0.65	0.74	0.66	0.65	0.64	0.66	0.66	0.62
SVM+bagging+ cost(1.5)	0.78	0.76	0.78	0.61	0.42	0.43	0.54	0.61	0.55	0.55	0.62	0.61
SVM+bagging+ cost(2.0)	0.86	0.81	0.82	0.59	0.27	0.32	0.46	0.54	0.48	0.51	0.58	0.55
SVM+bagging+ cost(2.5)	0.96	0.87	0.80	0.64	0.05	0.21	0.43	0.56	0.38	0.46	0.55	0.58
SVM+bagging+ cost(3.0)	1.00	0.95	0.79	0.64	0.00	0.04	0.40	0.53	0.36	0.39	0.53	0.57

G0—entire data set; 1331 patients with 476 worsening cases

G1—patients with initial EDSS < 2; 767 patients with 292 worsening cases

G2—patients with initial EDSS > = 2 and < 4; 429 patients with 147 worsening cases

G3—patients with initial EDSS > = 4; 135 patients with 37 worsening cases

Abbreviations: EDSS-Expanded Disability Status Scale, SVM-Support vector machines

Sensitivity is defined in this study as the proportion of subjects who worsen that are correctly classified. Specificity is defined as the proportion of subjects who did not worsen that are correctly classified. Overall accuracy is defined as the proportion of all subjects who are correctly classified.

### Prediction based on longitudinal clinical data

Given the poor accuracy using baseline data alone, we tested whether the inclusion of information available up to 1 year of follow-up (Tables 3 and 4) and up to 2 years of follow up (Tables 5 and 6) would improve our ability to predict a patient’s disease course at the 5 year mark. We observe that the overall accuracy improved slightly for all models with the inclusion of longitudinal data, but more importantly the accuracy of the progressive group improved (i.e., the model is no longer so heavily biased toward the majority class in the data). The difference between 1 year and 2 years of data was larger when the MRI data was omitted. Thus we conclude that the best option is to use 1 year of longitudinal data coupled with the MRI data. Table 7 compares accuracy rates for group 2 only (initial 2≥ EDSS <4) with 12 months versus 24 months of clinical and MRI data. For this subgroup, we conclude that longer follow up improved the accuracy of predicting non-progressive EDSS cases.

**Table 4:** Predictive accuracy for different groups using 12 month visit with MRI based on ten-fold cross-validation.

Method	Sensitivity				Specificity				Overall Accuracy			
	G0	G1	G2	G3	G0	G1	G2	G3	G0	G1	G2	G3
Logistic+bagging	0.67	0.64	0.52	0.50	0.68	0.67	0.64	0.59	0.68	0.66	0.60	0.57
logistic	0.55	0.58	0.37	0.48	0.78	0.73	0.66	0.63	0.69	0.66	0.57	0.60
SVM+bagging	0.71	0.72	0.75	0.48	0.68	0.67	0.66	0.59	0.69	0.69	0.69	0.56
SVM+bagging+ cost(1.5)	0.81	0.82	0.80	0.52	0.59	0.58	0.57	0.55	0.67	0.68	0.65	0.54
SVM+bagging+ cost(2.0)	0.85	0.85	0.81	0.48	0.53	0.54	0.50	0.54	0.65	0.67	0.60	0.53
SVM+bagging+ cost(2.5)	0.86	0.87	0.77	0.48	0.49	0.49	0.47	0.54	0.63	0.65	0.57	0.52
SVM+bagging+ cost(3.0)	0.86	0.87	0.79	0.52	0.47	0.46	0.45	0.60	0.62	0.63	0.56	0.58

G0—entire data set; 574 patients with 216 worsening cases

G1—patients with initial EDSS < 2; 368 patients with 153 worsening cases

G2—patients with initial EDSS > = 2 and < 4; 161 patients with 53 worsening cases

G3—patients with initial EDSS > = 4; 45 patients with 10 worsening cases

Abbreviations: EDSS-Expanded Disability Status Scale, SVM-Support vector machines

Sensitivity is defined in this study as the proportion of subjects who worsen that are correctly classified. Specificity is defined as the proportion of subjects who did not worsen that are correctly classified. Overall accuracy is defined as the proportion of all subjects who are correctly classified.

**Table 5:** Predictive accuracy for different groups using 24 month visit without MRI based on ten-fold cross-validation.

Method	Sensitivity				Specificity				Overall Accuracy			
	G0	G1	G2	G3	G0	G1	G2	G3	G0	G1	G2	G3
Logistic+bagging	0.62	0.60	0.60	0.60	0.70	0.69	0.69	0.71	0.67	0.66	0.66	0.67
logistic	0.47	0.47	0.51	0.57	0.86	0.81	0.81	0.74	0.73	0.69	0.72	0.70
SVM+bagging	0.61	0.58	0.64	0.54	0.79	0.77	0.77	0.62	0.73	0.71	0.73	0.60
SVM+bagging+ cost(1.5)	0.77	0.74	0.75	0.65	0.53	0.56	0.66	0.59	0.61	0.62	0.69	0.61
SVM+bagging+ cost(2.0)	0.81	0.77	0.76	0.63	0.45	0.43	0.60	0.60	0.57	0.55	0.65	0.60
SVM+bagging+ cost(2.5)	0.84	0.81	0.77	0.67	0.37	0.38	0.57	0.54	0.52	0.53	0.63	0.57
SVM+bagging+ cost(3.0)	0.87	0.82	0.75	0.68	0.3	0.33	0.58	0.59	0.49	0.51	0.63	0.61

G0—entire data set; 1236 patients with 409 worsening cases

G1—patients with initial EDSS < 2; 714 patients with 255 worsening cases

G2—patients with initial EDSS > = 2 and < 4; 397 patients with 122 worsening cases

G3—patients with initial EDSS > = 4; 125 patients with 32 worsening cases

Abbreviations: EDSS-Expanded Disability Status Scale, SVM-Support vector machines

Sensitivity is defined in this study as the proportion of subjects who worsen that are correctly classified. Specificity is defined as the proportion of subjects who did not worsen that are correctly classified. Overall accuracy is defined as the proportion of all subjects who are correctly classified.

**Table 6:** Predictive accuracy for different groups using 24 month visit data with MRI based on ten-fold cross-validation.

Method	Sensitivity				Specificity				Overall Accuracy			
	G0	G1	G2	G3	G0	G1	G2	G3	G0	G1	G2	G3
Logistic+bagging	**0.67**	0.59	0.51	0.46	0.70	0.66	0.61	0.64	0.69	0.63	0.58	0.60
logistic	0.59	0.57	0.51	0.58	0.78	0.70	0.73	0.61	0.71	0.65	0.66	0.61
SVM+bagging	0.65	0.65	0.74	0.30	0.74	0.74	0.76	0.46	0.71	0.70	0.75	0.43
SVM+bagging+ cost(1.5)	0.79	0.81	0.77	0.38	0.59	0.56	0.69	0.48	0.67	0.66	0.72	0.46
SVM+bagging+ cost(2.0)	0.81	0.82	0.82	0.40	0.56	0.54	0.62	0.45	0.65	0.65	0.68	0.44
SVM+bagging+ cost(2.5)	0.82	0.84	0.81	0.40	0.54	0.52	0.61	0.40	0.64	0.65	0.68	0.40
SVM+bagging+ cost(3.0)	0.82	0.84	0.80	0.28	0.51	0.48	0.61	0.41	0.63	0.63	0.67	0.38

G0—entire data set; 574 patients with 212 worsening cases

G1—patients with initial EDSS < 2; 370 patients with 151 worsening cases

G2—patients with initial EDSS > = 2 and < 4; 159 patients with 51 worsening cases

G3—patients with initial EDSS > = 4; 45 patients with 10 worsening cases

Abbreviations: EDSS-Expanded Disability Status Scale, SVM-Support vector machines

Sensitivity is defined in this study as the proportion of subjects who worsen that are correctly classified. Specificity is defined as the proportion of subjects who did not worsen that are correctly classified. Overall accuracy is defined as the proportion of all subjects who are correctly classified.

**Table 7:** Comparison of predictive accuracy for Group 2 (G2) using 12 month and 24 month clinical and MRI data based on ten-fold cross-validation.

cost	Accuracy of predicting worsening EDSS class (Sensitivity)		Accuracy of predicting non-worsening EDSS class (Specificity)		Overall Accuracy	
	1Y	2Y	1Y	2Y	1Y	2Y
1	0.75	0.74	0.66	0.76	0.69	0.75
1.5	0.80	0.77	0.57	0.69	0.65	0.72
2	0.81	0.82	0.50	0.62	0.60	0.68
2.5	0.77	0.81	0.47	0.61	0.57	0.68
3	0.79	0.80	0.45	0.61	0.56	0.67

### Prediction when mitigating for class imbalance

We next examined the difference in performance for all models when bagging + undersampling was used to improve performance for the minority class in the data (the progressive class). We show the results in Tables 3–6 for both 1 year and 2-year data for LR and Linear SVM. We observe that for a given longitudinal dataset, applying bagging improves performance for the progressive class for both methods (e.g., for LR and 12 months of data the accuracy for the progressive class was 67% for the bagging model and 55% for the model built without bagging). Indeed, not applying bagging to handle the class imbalance, results in slightly higher overall accuracy, but significantly lowers the accuracy for the progressive class which is the more costly of the two in terms of misclassification errors. This occurs because logistic regression and SVM minimize overall error and thus are skewed by the much larger number of non-progressive patients in the data.

### Misclassification costs

In addition, we studied varied misclassification costs in each of the subgroups within the SVM analysis. Results are included in Tables 3–6. The overall predictive accuracy with uniform misclassification costs with SVM was 69%. Note that we applied bagging in building all models. With this equal misclassification cost, the accuracy of predicting progressive and non-progressive cases was largely similar with SVM. When the cost of false negatives was increased relative to the cost of false positives (cost = 1.5), the accuracy of correctly identifying progressive EDSS cases increased to 81% and 79% for the 1-year and 2 year data respectively with a decrease in the accuracy of the non-progressive class to 59% for both data sets. Further increases in the relative cost of false positives led to small increases in the accuracy of the progressive class at the expense of lower accuracy in the non-progressive class and lower overall accuracy.

### Features predictive of 5 year outcome from SVM models

We evaluated the predictive power of each feature, and as an example, we show predictive features for G1 at cost = 1.5 for progressive and non-progressive cases using one year information, with and without MRI data (S1 and S2 Tables). Because we applied a linear SVM, we can rank the predictors by the magnitude of their weight, giving us a rough estimate of their ability to predict progression. Because our experiments are based on a 10-fold cross-validation, for each predictor we show the average rank for each group. Note that we show these values for each class separately because in the process of classifying a patient, each feature will either contribute positively (for progressive class) or negatively (for non-progressive class). Ranking these contributions separately allows us to gain insights into the features that are most pertinent to each class. Examining the clinical features alone, the top 20 predictors of both progressive and non-progressive cases included EDSS score, disease activity score, sensory, cerebellar, visual, mental, bowel/bladder and brainstem FS scores. However race, ethnicity and family history of MS, appeared as predictors of non-progressive cases but not of progressive cases. When MRI information was included, BPF was in the top 20 of the predictors of non-progressive cases, but not of progressive cases. In contrast, T2 lesion volume appeared within the top 50 predictors of progressive cases, but did not appear within the non-progressive cases. Similar trends in predictive features were seen with 24-month data, including the observation that BPF appeared in the top 50 predictors of non-progressive cases exclusively, while T2 lesion volume appeared exclusively as a predictor of progressive cases (S1 and S2 Tables).

## Discussion

In this study, machine-learning techniques including SVM with bagging/undersampling and cost misclassification were used to assess the ability of clinical and MRI features to predict EDSS status at up to five years and compared to logistic regression. Using baseline clinical data only and treating false positives and false negatives equally, the models had accuracies that were better than random guessing but were heavily skewed toward predicting all patients were non-progressive. The skew improved slightly when baseline MRI data were included. Further follow-up observation at 1 year improved overall accuracy with improvements in predicting non-progressive cases as did bagging coupled with undersampling to address the class imbalance in the training data. When non-uniform misclassification costs were included in the SVM model, there was a still larger improvement in predicting progressive cases, however with expense of decreased accuracy in predicting non-progressive cases.

Predictors of disease course using both 1 year and 2-year data included clinical features. Demographic features of race, ethnicity and family history of MS ranked more highly as predictors of non-progressive MS. Inclusion of MRI predictors revealed that BPF ranked highly as a predictor of non-progressive MS but not of progressive cases, while T2 lesion volume predicted progressive cases.

Although several studies in predicting disease course in MS have been undertaken using logistic regression,[17] Markov modeling,[18–20] and more recently a Bayesian modeling approach,[21] there has been limited exploration of machine-learning techniques in MS. One study has explored a neural network computational classifier in 51 MS patients to predict disease course.[22] Whether an accuracy of >70% is considered a reasonable benchmark for machine-learning, depends on the context and condition to which the method is being applied. In our case it may be argued that it is more critical to detect progressive cases accurately without a large number of false positives, in order to place these patients on more aggressive treatments. And therefore, using SVM with a cost of 1.5, predictive accuracy of 81% on progressive with 59% on non-progressive may be clinically acceptable to most. However, this balance needs to be determined by individual physicians and patients.

To our knowledge, ours is the first study to apply LR and SVM models to a large cohort over 1600 subjects, using multiple clinical and MRI predictors. In addition the incorporation unequal weighting of classification errors provide a novel approach to improving prediction in the group of interest, which in this case is correctly predicting the progressive group. The use of misclassification costs in SVM models introduces a new paradigm into modeling disease outcomes, which may more closely mimic daily decision-making. Extensions of this approach may be tailored to individual physician styles of practice, or patient types, including degree of risk-taking and tolerance of side effects.

Clinical observation for at least one year was required to obtain a >80% accuracy in predicting progressive cases, suggesting that early changes are crucial to subsequent disease course. Amongst the clinical predictors of disease course, race, ethnicity and family history of MS were highly ranked in predicting the non-progressive group, raising the possibility that inclusion of additional genetic and environmental features may further improve accuracy in this group.

BPF ranked highly as a predictor of non-progressive status, consistent with previous findings.[23–25] In contrast the T2 lesion volume ranked highly amongst predictors of progressive patients, indicating that accrual of lesions, and possibly relapses plays an important role in short-term and possibly long-term disability accrual as has been shown in other studies.[26–28]. These findings support the concept that BPF and T2 lesions provide complementary information about different disease processes in MS.

There were several limitations to our study. Firstly, in order to establish the models, we focused largely on clinical data points and quantitative MRI features. Further work plans to incorporate additional biomarker data. Second, there are limitations to our outcome measure of progressive or non-progressive cases based on change in EDSS values over 5 years. The EDSS scale has been criticized as a somewhat insensitive scale, particularly to visual and cognitive decline. Further studies incorporating these measures should be explored. Treatment fields were not included as predictors in this study, because our goal was to first establish the effect of early clinical and MRI markers in association with disease course. Further studies will explore the effects of treatments. Lastly, for machine learning methods, the size of training samples is essential for the quality of the classifier. One of our patient subgroups G3 (EDSS≥4) was too small to obtain accurate predictions. Larger, collaborative studies may be required to accurately predict disease course in subgroups with limited subject numbers.

Our results require validation in additional datasets. Future work may focus on incorporating additional features including additional neuroimaging measures, blood and genetic biomarkers. Development of joint physician-patient visualization and decision-making tools, as have been discussed in other works,[29–32] may be further enabled using predictive algorithms discussed here. Machine learning techniques, and in particular SVM may be powerful tools for the personalization of MS therapeutic approaches.

## Supporting information

S1 Table Predictors of 5 year outcomes in G1, 1Y, cost = 1 (top 50 shown). (DOCX)

S2 Table Predictors of 5 year outcomes in G1, 2Y, cost = 1 (top 50 shown). (DOCX)
