# Supplementary material for: Exploration of machine learning techniques in predicting multiple sclerosis disease course
Source: PLoS One. 2017 Apr 5;12(4):e0174866. doi: 10.1371/journal.pone.0174866 (PMC5381810; doi:10.1371/journal.pone.0174866)
Supplement: S1 Table — (DOCX) [file pone.0174866.s001.docx]

**Supplemental Tables:**

**S1 Table – Predictors of 5 year outcomes in G1, 1Y, cost =1 (top 50 shown)**

| **Rank** | **non-progressive (without MRI)** | **progressive (without MRI)** |
| --- | --- | --- |
| **1** | **'EDSS_0m** | **'EDSS_diff_12m-0m** |
| **2** | **'DISEASE_ACTIVITY_6m=1** | **'PYRAMIDAL_FUNCTION_12m** |
| **3** | **'EDSS_6m** | **'BOWEL_BLADDER_FUNCTION_12m** |
| **4** | **'DISEASE_ACTIVITY_0m=1** | **'EDSS_diff_6m-0m** |
| **5** | **'SENSORY_FUNCTION_0m** | **'SENSORY_FUNCTION_diff_12m-0m** |
| **6** | **'SENSORY_FUNCTION_6m** | **'AI_6m** |
| **7** | **'BOWEL_BLADDER_FUNCTION_diff_6m-0m** | **'PYRAMIDAL_FUNCTION_diff_12m-0m** |
| **8** | **'DISEASE_ACTIVITY_12m=1** | **'AI_12m** |
| **9** | **'BRAINSTEM_FUNCTION_6m** | **'AI_0m** |
| **10** | **'VISUAL_FUNCTION_0m** | **'VISUAL_FUNCTION_diff_12m-0m** |
| **11** | **'RACE=6** | **'BOWEL_BLADDER_FUNCTION_0m** |
| **12** | **'DISEASE_ACTIVITY_12m=3** | **'DISEASE_ACTIVITY_0m=2** |
| **13** | **'BRAINSTEM_FUNCTION_0m** | **'MENTAL_FUNCTION_diff_12m-0m** |
| **14** | **'FAMILY_MS=3** | **'VISUAL_FUNCTION_diff_6m-0m** |
| **15** | **'SENSORY_FUNCTION_12m** | **'EDSS_12m** |
| **16** | **'RACE=3** | **'DISEASE_ACTIVITY_6m=6** |
| **17** | **'ETHNICITY=3** | **'BRAINSTEM_FUNCTION_diff_12m-0m** |
| **18** | **'DISEASE_STEP_diff_6m-0m** | **'CEREBELLAR_FUNCTION_diff_6m-0m** |
| **19** | **'CEREBELLAR_FUNCTION_0m** | **'DISEASE_STEP_12m** |
| **20** | **'MENTAL_FUNCTION_6m** | **'MENTAL_FUNCTION_12m** |
| **21** | **'BRAINSTEM_FUNCTION_diff_6m-0m** | **'DISEASE_ACTIVITY_0m=5** |
| **22** | **'DISEASE_ACTIVITY_0m=6** | **'BOWEL_BLADDER_FUNCTION_diff_12m-0m** |
| **23** | **'MENTAL_FUNCTION_0m** | **'DISEASE_ACTIVITY_6m=3** |
| **24** | **'ETHNICITY=1** | **'FAMILY_MS=2** |
| **25** | **'PYRAMIDAL_FUNCTION_diff_6m-0m** | **'CEREBELLAR_FUNCTION_diff_12m-0m** |
| **26** | **'FAMILY_MS=1** | **'SENSORY_FUNCTION_diff_6m-0m** |
| **27** | **'SMOKING_EVER** | **'DISEASE_ACTIVITY_6m=5** |
| **28** | **'DISEASE_STEP_6m** | **'DISEASE_STEP_diff_12m-0m** |
| **29** | **'MENTAL_FUNCTION_diff_6m-0m** | **'CEREBELLAR_FUNCTION_6m** |
| **30** | **'RACE=1** | **'DISEASE_ACTIVITY_12m=2** |
| **31** | **'DISEASE_ACTIVITY_12m** | **'VISUAL_FUNCTION_12m** |
| **32** | **'BOWEL_BLADDER_FUNCTION_6m** | **'VISIT_AGE'** |
| **33** | **'AI_diff_12m-0m** | **'BRAINSTEM_FUNCTION_12m** |
| **34** | **'DISEASE_ACTIVITY_0=7** | **'CEREBELLAR_FUNCTION_12m** |
| **35** | **'DISEASE_ACTIVITY_12m=6** | **'DISEASE_ACTIVITY_0m=3** |
| **36** | **'DISEASE_STEP_0m** | **'RACE=4** |
| **37** | **'AI_6_diff_6m-0m** | **'ETHNICITY=2** |
| **38** | **'DISEASE_ACTIVITY_6m=2** | **'DISEASE_ACTIVITY_12m=7** |
| **39** | **'DISEASE_ACTIVITY_12m=4** | **'SEX'** |
| **40** | **'DISEASE_ACTIVITY_6m=7** | **'PYRAMIDAL_FUNCTION_0m** |
| **41** | **'PYRAMIDAL_FUNCTION_6m** | **'VISUAL_FUNCTION_6m** |
| **42** | **'VISUAL_FUNCTION_6m** | **'AI_diff_6m-0m** |
| **43** | **'DISEASE_ACTIVITY_0m=3** | **'PYRAMIDAL_FUNCTION_6m** |
| **44** | **'SEX'** | **'RACE=2** |
| **45** | **'CEREBELLAR_FUNCTION_12m** | **'DISEASE_ACTIVITY_6m=4** |
| **46** | **'RACE=8** | **'AI_diff_12m-0m** |
| **47** | **'PYRAMIDAL_FUNCTION_0m** | **'DISEASE_ACTIVITY_12m=5** |
| **48** | **'BRAINSTEM_FUNCTION_12m** | **'DISEASE_STEP_0m** |
| **49** | **'RACE=7** | **'RACE=7** |
| **50** | **'CEREBELLAR_FUNCTION_6m** | **'BOWEL_BLADDER_FUNCTION_6m** |

| **Rank** | **non-progressive (with MRI)** | **progressive (with MRI)** |
| --- | --- | --- |
| **1** | **'EDSS_0m** | **'EDSS_diff_12m-0m** |
| **2** | **'EDSS_6m** | **'EDSS_diff_6m-0m** |
| **3** | **'DISEASE_ACTIVITY_0m=1** | **'PYRAMIDAL_FUNCTION_12m** |
| **4** | **'DISEASE_ACTIVITY_6m=1** | **'PYRAMIDAL_FUNCTION_diff_12m-0m** |
| **5** | **'RACE=6** | **'DISEASE_ACTIVITY_0m=2** |
| **6** | **'DISEASE_ACTIVITY_12m=3** | **'MENTAL_FUNCTION_diff_12m-0m** |
| **7** | **'EDSS_12m** | **'CEREBELLAR_FUNCTION_diff_12m-0m** |
| **8** | **'BRAINSTEM_FUNCTION_6m** | **'AI_6** |
| **9** | **'BPF_12m** | **'MENTAL_FUNCTION_12m** |
| **10** | **'DISEASE_ACTIVITY_0m=6** | **'AI_12m** |
| **11** | **'BRAINSTEM_FUNCTION_diff_6m-0m** | **'DISEASE_STEP_6m'** |
| **12** | **'BOWEL_BLADDER_FUNCTION_6m** | **'DISEASE_ACTIVITY_12m=5** |
| **13** | **'FAMILY_MS=1** | **'SENSORY_FUNCTION_diff_12m-0m** |
| **14** | **'BPF_6m** | **'SENSORY_FUNCTION_diff_6m-0m** |
| **15** | **'BRAINSTEM_FUNCTION_0m** | **'VISUAL_FUNCTION_diff_12m-0m** |
| **16** | **'BOWEL_BLADDER_FUNCTION_diff_6m-0m** | **'CEREBELLAR_FUNCTION_12m** |
| **17** | **'ETHNICITY=3** | **'CEREBELLAR_FUNCTION_diff_6m-0m** |
| **18** | **'CEREBELLAR_FUNCTION_0m'** | **'VISUAL_FUNCTION_diff_6m-0m** |
| **19** | **'SENSORY_FUNCTION_0m'** | **'DISEASE_ACTIVITY_12m=7** |
| **20** | **'BPF_0m'** | **'AI_0m'** |
| **21** | **'BPF_diff_12m-0m** | **'DISEASE_STEP_diff_6m-0m** |
| **22** | **'BRAINSTEM_FUNCTION_12m** | **'DISEASE_STEP_12m'** |
| **23** | **'VISUAL_FUNCTION_0m** | **'RACE=7'** |
| **24** | **'BPF_diff_6m-0m** | **'VISUAL_FUNCTION_6m** |
| **25** | **'DISEASE_ACTIVITY_12m=1** | **'RACE=3'** |
| **26** | **'MENTAL_FUNCTION_0m** | **'VISUAL_FUNCTION_12** |
| **27** | **'BOWEL_BLADDER_FUNCTION_0m** | **'AI_diff_6m-0m** |
| **28** | **'ETHNICITY=2** | **'ETHNICITY=1'** |
| **29** | **'PYRAMIDAL_FUNCTION_diff_6m-0m** | **'LESION_VOLUME_12m** |
| **30** | **'PYRAMIDAL_FUNCTION_6m** | **'DISEASE_ACTIVITY_6m=6** |
| **31** | **'DISEASE_ACTIVITY_12m=6** | **'FAMILY_MS=2** |
| **32** | **'SMOKING_EVER'** | **'DISEASE_ACTIVITY_6m=2** |
| **33** | **'MENTAL_FUNCTION_6m** | **'DISEASE_ACTIVITY_0m=7** |
| **34** | **'FAMILY_MS=3** | **'SEX'** |
| **35** | **'PYRAMIDAL_FUNCTION_0m** | **'DISEASE_STEP_diff_12m-0m** |
| **36** | **'DISEASE_ACTIVITY_6m=5** | **'CEREBELLAR_FUNCTION_6m** |
| **37** | **'BOWEL_BLADDER_FUNCTION_12m** | **'LESION_VOLUME_6m** |
| **38** | **'DISEASE_ACTIVITY_0m=3** | **'VISIT_AGE'** |
| **39** | **'DISEASE_ACTIVITY_6m=3** | **'SENSORY_FUNCTION_12m** |
| **40** | **'DISEASE_ACTIVITY_0m=5** | **'MENTAL_FUNCTION_diff_6m-0m** |
| **41** | **'RACE=5** | **'LESION_VOLUME_diff_12m-0m** |
| **42** | **'BRAINSTEM_FUNCTION_diff_12m-0m** | **'SENSORY_FUNCTION_6m** |
| **43** | **'BOWEL_BLADDER_FUNCTION_diff_12m-0m** | **'BOWEL_BLADDER_FUNCTION_diff_12m-0m** |
| **44** | **'DISEASE_STEP_0m** | **'DISEASE_STEP_0m** |
| **45** | **'AI_diff_12m-0m** | **'AI_diff_12m-0m** |
| **46** | **'SENSORY_FUNCTION_6m** | **'PYRAMIDAL_FUNCTION_0m** |
| **47** | **'DISEASE_ACTIVITY_12m=2** | **'DISEASE_ACTIVITY_0m=4** |
| **48** | **'MENTAL_FUNCTION_diff_6m-0m** | **'DISEASE_ACTIVITY_6m=7** |
| **49** | **'SENSORY_FUNCTION_12m** | **'LESION_VOLUME_diff_6m-0m** |
| **50** | **'LESION_VOLUME_0m** | **'DISEASE_ACTIVITY_0m=3** |

Abbreviations:

AI – Ambulation index

BPF – brain parenchymal fraction

EDSS – Expanded disability status scale

Disease activity categories:

1 stable

2 active - current attack

3 active - attack in past 6 months

4 active - progressive

5 improved

6 active - MRI activity only

7 active - MRI plus attack-progression

Race:

1 American Indian or Alaska Native

2 Asian

3 Black or African American

5 More than one race

6 Native Hawaiian or Other Pacific Islander

7 White

8 Unknown or not reported

Ethnicity:

1 Hispanic or latino

2 Not hispanic or latino

3 Unknown

*Red highlighted variables show key MRI features
