# Supplementary material for: Exploration of machine learning techniques in predicting multiple sclerosis disease course
Source: PLoS One. 2017 Apr 5;12(4):e0174866. doi: 10.1371/journal.pone.0174866 (PMC5381810; doi:10.1371/journal.pone.0174866)
Supplement: S2 Table — (DOCX) [file pone.0174866.s002.docx]

**S2 Table - Predictors of 5 year outcomes in G1, 2Y, cost =1 (top 50 shown)**

| **Rank** | **non-progressive (without MRI)** | **progressive (without MRI)** |
| --- | --- | --- |
| **1** | **'EDSS_0m** | **'EDSS_diff_24m-0m** |
| **2** | **'EDSS_6m'** | **'EDSS_24m** |
| **3** | **'DISEASE_ACTIVITY_6m=1** | **'EDSS_diff_18m-0m** |
| **m4** | **'DISEASE_ACTIVITY_0m=1** | **'CEREBELLAR_FUNCTION_diff_24m-0m** |
| **5** | **'DISEASE_ACTIVITY_12m=3** | **'MENTAL_FUNCTION_24m** |
| **6** | **'BRAINSTEM_FUNCTION_24m** | **'PYRAMIDAL_FUNCTION_12m** |
| **7** | **'EDSS_12m** | **'SENSORY_FUNCTION_diff_18m-0m** |
| **8** | **'DISEASE_ACTIVITY_12m=1** | **'MENTAL_FUNCTION_diff_24m-0m** |
| **9** | **'SENSORY_FUNCTION_0m** | **'PYRAMIDAL_FUNCTION_diff_12m-0m** |
| **10** | **'BRAINSTEM_FUNCTION_6m** | **'CEREBELLAR_FUNCTION_24m** |
| **11** | **'BOWEL_BLADDER_FUNCTION_diff_6m-0m** | **'SENSORY_FUNCTION_diff_12m-0m** |
| **12** | **'ETHNICITY=3** | **'AI_24m** |
| **13** | **'DISEASE_STEP_diff_24m-0m** | **'DISEASE_ACTIVITY_12m=2** |
| **14** | **'VISUAL_FUNCTION_0m** | **'VISUAL_FUNCTION_diff_12m=0** |
| **15** | **'BRAINSTEM_FUNCTION_0m** | **'CEREBELLAR_FUNCTION_diff_6m-0m** |
| **16** | **'BOWEL_BLADDER_FUNCTION_6m** | **'BRAINSTEM_FUNCTION_diff_12m-0m** |
| **17** | **'RACE=6'** | **'DISEASE_ACTIVITY_0m=2** |
| **18** | **'BRAINSTEM_FUNCTION_18m** | **'PYRAMIDAL_FUNCTION_18m** |
| **19** | **'MENTAL_FUNCTION_diff_12m-0m** | **'AI_12m** |
| **20** | **'DISEASE_STEP_24m** | **'BOWEL_BLADDER_FUNCTION_12m** |
| **21** | **'FAMILY_MS=2** | **'AI_6m** |
| **22** | **'SENSORY_FUNCTION_24m** | **'DISEASE_ACTIVITY_6m=2** |
| **23** | **'MENTAL_FUNCTION_12m** | **'ETHNICITY=2** |
| **24** | **'SENSORY_FUNCTION_6m** | **'PYRAMIDAL_FUNCTION_diff_18m-0m** |
| **25** | **'DISEASE_ACTIVITY_24m=6** | **'EDSS_diff_12m-0m** |
| **26** | **'BRAINSTEM_FUNCTION_diff_24m-0m** | **'AI_18m** |
| **27** | **'MENTAL_FUNCTION_diff_6m-0m** | **'AI_24_diff_24m-0m** |
| **28** | **'CEREBELLAR_FUNCTION_0m** | **'CEREBELLAR_FUNCTION_6m** |
| **29** | **'PYRAMIDAL_FUNCTION_diff_24m-0m** | **'DISEASE_ACTIVITY_6m=6** |
| **30** | **'SMOKING_EVER'** | **'AI_0m** |
| **31** | **'DISEASE_ACTIVITY_0m=6'** | **'BOWEL_BLADDER_FUNCTION_diff_12m-0m** |
| **32** | **'CEREBELLAR_FUNCTION_18m** | **'VISUAL_FUNCTION_diff_24m-0m** |
| **33** | **'MENTAL_FUNCTION_6m** | **'DISEASE_ACTIVITY_12m=5** |
| **34** | **'VISUAL_FUNCTION_6m** | **'SENSORY_FUNCTION_diff_6m-0m** |
| **35** | **'DISEASE_ACTIVITY_18m=6** | **'DISEASE_ACTIVITY_0m=5** |
| **36** | **'DISEASE_ACTIVITY_24m=5** | **'MENTAL_FUNCTION_18m** |
| **37** | **'PYRAMIDAL_FUNCTION_24m** | **'SENSORY_FUNCTION_18m** |
| **38** | **'VISUAL_FUNCTION_18m** | **'FAMILY_MS=3** |
| **39** | **'BOWEL_BLADDER_FUNCTION_diff_24m-0m** | **'EDSS_18m** |
| **40** | **'DISEASE_ACTIVITY_24m=8** | **'VISUAL_FUNCTION_diff_18m-0m** |
| **41** | **'DISEASE_STEP_diff_18m-0m** | **'DISEASE_ACTIVITY_12m=7** |
| **42** | **'BRAINSTEM_FUNCTION_diff_6m-0m** | **'VISIT_AGE'** |
| **43** | **'BOWEL_BLADDER_FUNCTION_diff_18m-0m** | **'DISEASE_ACTIVITY_24m=4** |
| **44** | **'PYRAMIDAL_FUNCTION_diff_6m-0m** | **'DISEASE_ACTIVITY_18m=5** |
| **45** | **'DISEASE_STEP_diff_6m-0mm** | **'BRAINSTEM_FUNCTION_12** |
| **46** | **'DISEASE_ACTIVITY_18m=7** | **'SENSORY_FUNCTION_diff_24m-0m** |
| **47** | **'CEREBELLAR_FUNCTION_12m** | **'MENTAL_FUNCTION_diff_18m-0m** |
| **48** | **'BRAINSTEM_FUNCTION_diff_18m-0m** | **'RACE=7** |
| **49** | **'DISEASE_ACTIVITY_18m=4** | **'DISEASE_ACTIVITY_0m=3** |
| **50** | **'DISEASE_STEP_diff_12m-0m** | **'SEX'** |

| **Rank** | **non-progressive (with MRI)** | **progressive (with MRI)** |
| --- | --- | --- |
| **1** | **'EDSS_0m** | **'EDSS_diff_24m-0m** |
| **2** | **'EDSS_6m** | **'EDSS_diff_18m-0m** |
| **3** | **'DISEASE_ACTIVITY_12m=3** | **'EDSS_diff_12m-0m** |
| **4** | **'DISEASE_ACTIVITY_6m=1** | **'MENTAL_FUNCTION_24m** |
| **5** | **'RACE=6** | **'MENTAL_FUNCTION_diff_24m-0m** |
| **6** | **'BRAINSTEM_FUNCTION_6m** | **'AI_24m'** |
| **7** | **'DISEASE_ACTIVITY_0m=1** | **'SENSORY_FUNCTION_diff_18m-0m** |
| **8** | **'EDSS_12m** | **'PYRAMIDAL_FUNCTION_diff_12m-0m** |
| **9** | **'AI_diff_18m-0m** | **'SENSORY_FUNCTION_diff_12m-0m** |
| **10** | **'SENSORY_FUNCTION_24m** | **'AI_6m'** |
| **11** | **'FAMILY_MS=1** | **'EDSS_diff_6m-0m** |
| **12** | **'SENSORY_FUNCTION_0m** | **'CEREBELLAR_FUNCTION_diff_12m-0m** |
| **13** | **'BOWEL_BLADDER_FUNCTION_6m** | **'PYRAMIDAL_FUNCTION_12m** |
| **14** | **'BRAINSTEM_FUNCTION_24m** | **'CEREBELLAR_FUNCTION_diff_6m-0m** |
| **15** | **'BRAINSTEM_FUNCTION_0m** | **'CEREBELLAR_FUNCTION_diff_24m-0m** |
| **16** | **'VISUAL_FUNCTION_0m** | **'SENSORY_FUNCTION_diff_6m-0m** |
| **17** | **'CEREBELLAR_FUNCTION_0m** | **'LESION_VOLUME_24m** |
| **18** | **'EDSS_18m** | **'AI_24_ 24m-0'm** |
| **19** | **'BOWEL_BLADDER_FUNCTION_diff_6m-0m** | **'AI_12m** |
| **20** | **'BRAINSTEM_FUNCTION_diff_6m-0m** | **'SENSORY_FUNCTION_18m** |
| **21** | **'BRAINSTEM_FUNCTION_12m** | **'AI_0m** |
| **22** | **'BRAINSTEM_FUNCTION_18m** | **'VISUAL_FUNCTION_diff_18m-0m** |
| **23** | **'PYRAMIDAL_FUNCTION_6m** | **'DISEASE_ACTIVITY_24m=1** |
| **24** | **'DISEASE_ACTIVITY_24m=3** | **'DISEASE_ACTIVITY_6m=2'** |
| **25** | **'DISEASE_ACTIVITY_24m=5** | **'CEREBELLAR_FUNCTION_12m'** |
| **26** | **'DISEASE_STEP_24m** | **'LESION_VOLUME_diff_24m-0m** |
| **27** | **'DISEASE_ACTIVITY_18m=6** | **'DISEASE_ACTIVITY_0m=2'** |
| **28** | **'DISEASE_STEP_diff_24m=0** | **'DISEASE_ACTIVITY_12m=2'** |
| **29** | **'BPF_24m** | **'AI_diff_6m-0m** |
| **30** | **'BPF_18m** | **'VISUAL_FUNCTION_diff_12m-0m** |
| **31** | **'MENTAL_FUNCTION_6m** | **'RACE=7'** |
| **32** | **'BOWEL_BLADDER_FUNCTION_24m** | **'VISUAL_FUNCTION_diff_24m-0m** |
| **33** | **'VISUAL_FUNCTION_6m** | **'DISEASE_STEP_18m** |
| **34** | **'BPF_12m** | **'VISIT_AGE'** |
| **35** | **'DISEASE_ACTIVITY_18m=1** | **'CEREBELLAR_FUNCTION_diff_18m-0m** |
| **36** | **'SMOKING_EVER'** | **'DISEASE_STEP_diff_18m-0m** |
| **37** | **'BPF_diff_24m-0m** | **'DISEASE_ACTIVITY_12m=5** |
| **38** | **'BPF_diff_18m-0m** | **'EDSS_24m** |
| **39** | **'PYRAMIDAL_FUNCTION_0m'** | **'DISEASE_STEP_diff_6m-0m** |
| **40** | **'BPF_6m'** | **'MENTAL_FUNCTION_diff_18m-0m** |
| **41** | **'MENTAL_FUNCTION_diff_6m-0m'** | **'CEREBELLAR_FUNCTION_24m'** |
| **42** | **'BOWEL_BLADDER_FUNCTION_0m'** | **'DISEASE_STEP_6m'** |
| **43** | **'ETHNICITY=2'** | **'BOWEL_BLADDER_FUNCTION_diff_18m-0m** |
| **44** | **'BOWEL_BLADDER_FUNCTION_diff_24m-0m** | **'SENSORY_FUNCTION_12m'** |
| **45** | **'DISEASE_ACTIVITY_12m=1** | **'MENTAL_FUNCTION_18m** |
| **46** | **'MENTAL_FUNCTION_0m** | **'PYRAMIDAL_FUNCTION_diff_18m-0m** |
| **47** | **'AI_18m** | **'FAMILY_MS=2'** |
| **48** | **'PYRAMIDAL_FUNCTION_diff_6m-0m** | **'CEREBELLAR_FUNCTION_6m'** |
| **49** | **'BPF_0m** | **'DISEASE_ACTIVITY_18m=3'** |
| **50** | **'LESION_VOLUME_diff_12m-0m** | **'LESION_VOLUME_18m'** |

*Red highlighted variables show key MRI features
